# Implementation of the World Health Organization's QualityRights initiative in Ghana: an overview

**DOI:** 10.1192/bjo.2024.11

**Published:** 2024-05-13

**Authors:** Akwasi O. Osei, Caroline Amissah, Samuel Cudjoe Hanu, Priscilla E. Tawiah, Kwaku A. Brobbey, Yaw Amankwah Arthur, Joana Ansong, Sally-Ann Ohene, Leveana Gyimah, Humphrey Kofie, Daniel Taylor, Peter Badimak Yaro, Michelle Funk, Natalie Drew, Maria Francesca Moro, Mauro Giovanni Carta, Florence Kamayonza Baingana, Victus Kwaku Kpesese, Martin Orrell, Celline Cole, WHO QualityRights Ghana Team

**Affiliations:** Mental Health Authority, Ministry of Health, Accra, Ghana; Research Unit, Mental Health Authority, Ministry of Health, Accra, Ghana; Community Care Unit, Mental Health Authority, Ministry of Health, Accra, Ghana; Communications Department, Mental Health Authority, Ministry of Health, Accra, Ghana; Health Promotion Unit, Mental Health Authority, Ministry of Health, Accra, Ghana; Communicable Noncommunicable Disease Cluster, World Health Organization, Accra, Ghana; Mental Health Society of Ghana, Accra, Ghana; MindFreedom, Accra, Ghana; BasicNeeds, Tamale, Ghana; Mental Health Policy & Service Development, Department of Mental Health and Substance Abuse, World Health Organization Headquarters, Geneva, Switzerland; Irving Medical Center, Columbia University, USA; University of Cagliari, Italy; World Health Organization African Region, Brazaville, Congo; Administration, Mental Health Authority, Ministry of Health, Accra, Ghana; School of Health Sciences, Institute of Mental Health, University of Nottingham, UK; World Health Organization Headquarters, Geneva, Switzerland

**Keywords:** WHO QualityRights, mental health, human rights, cognitive, intellectual, psychosocial disability, Ghana

## Abstract

**Background:**

Globally, human rights violations experienced by persons with psychosocial, intellectual or cognitive disabilities continue to be a concern. The World Health Organization's (WHO) QualityRights initiative presents practical remedies to address these abuses. This paper presents an overview of the implementation of the initiative in Ghana.

**Aims:**

The main objective of the QualityRights initiative in Ghana was to train and change attitudes among a wide range of stakeholders to promote recovery and respect for human rights for people with psychosocial, intellectual and cognitive disabilities.

**Method:**

Reports of in-person and online training, minutes of meetings and correspondence among stakeholders of the QualityRights initiative in Ghana, including activities of international collaborators, were analysed to shed light on the implementation of the project in Ghana.

**Results:**

In-person and online e-training on mental health were conducted. At the time of writing, 40 443 people had registered for the training, 25 416 had started the training and 20 865 people had completed the training and obtained a certificate. The team conducted 27 in-person training sessions with 910 people. The successful implementation of the project is underpinned by a committed partnership among stakeholders, strong leadership from the coordinating agency, the acceptance of the initiative and the outcome. A few challenges, both in implementation and acceptance, are discussed.

**Conclusions:**

The exposure of the WHO QualityRights initiative to a substantial number of key stakeholders involved in mental healthcare in Ghana is critical to reducing human rights abuses for people with psychosocial, intellectual and cognitive disabilities.

Globally, the movement to transform the delivery of mental healthcare and to change attitudes toward people with psychosocial, intellectual or cognitive disabilities is gaining momentum.^1^ The Convention on the Rights of Persons with Disabilities (CRPD), which came into effect in 2008 and has been ratified by 185 states and the European Union, sets obligations on States Parties to promote the rights of people with disabilities on an equal basis with others, which requires significant changes in attitudes and practices, including in mental healthcare.^1^

Over the past several years, Portugal and Brazil led three important resolutions, adopted by the United Nations Human Rights Council, calling on member states and other United Nations agencies to take steps to end the widespread human rights violations, such as discrimination, social exclusion, involuntary treatment and coercive practices, that fail to respect the dignity of people living with psychosocial, intellectual or cognitive disabilities seen in mental health practice.^2–4^

Furthermore, the United Nations High Commissioner for Human Rights issued reports on mental health and human rights in 2017,^5,6^ which advocate for ending violations against people with psychosocial disabilities and recommend a number of policy shifts to fully realise their rights and recognise their autonomy, agency and dignity.

Subsequently, the United Nations Special Rapporteur on the Right to Health issued a landmark report^6^ to support the call by the Human Rights Council^3^ and High Commissioner for Human Rights,^5^ and recommended the global implementation of the World Health Organization's (WHO) QualityRights initiative. The report clearly stated that promoting and protecting human rights in mental healthcare requires more than legislative changes, and that a fundamental change is needed in the way psychiatry is practiced.^7^ The QualityRights initiative provides a framework to achieve this, along with clear guidance and practical tools for countries to realise such a change. According to Funk and Drew,^1^ the four key areas of work of the QualityRights initiative are:

- building the capacity of different stakeholders to combat stigma and discrimination, and to implement a human rights-based approach aligned with the CRPD;
- supporting countries to develop community-based mental health services that respect human rights, and use a person-centred recovery approach;
- supporting civil society to empower people with lived experience and others to conduct advocacy, specifically to integrate a human rights-based approach, and to influence policy;
- supporting governments to reform policy and legislation in line with the CRPD and other international human rights standards.

In Ghana, there have been reported widespread human rights abuses in terms of involuntary admissions and abusive treatment in some psychiatric facilities^8^ in recent years, including ‘state-sponsored human rights abuses’.^9^ It was against this background that the Mental Health Authority of Ghana (MHA) and other local partners collaborated with the WHO to implement the QualityRights initiative in Ghana to promote quality and human rights in mental healthcare in the country.

Ghana was the first country worldwide to adopt and implement WHO QualityRights initiative on a national basis. Other countries had implemented it on a smaller scale; for example, the state of Gujarat in India had first rolled out the initiative at the state level. Tunisia applied it at one hospital where face-to-face training was conducted. Previous studies on the initiative by Carta et al^10^ in Tunisia, and Morrissey^11^ in Iceland, found that the face-to-face training using the QualityRights training materials was effective in improving the knowledge and attitudes of participants toward a rights-based approach to mental health. The positive impact of the initiative was also confirmed in the research trial undertaken in public mental health facilities as part of the QualityRights initiative in Gujarat.^12^

One key focus of implementation of the initiative in Ghana has been training on human rights, which is the subject of this paper.

Here, we provide a description of the inception and planning phases of the project, including the identification of stakeholders and partners in implementing the project. Thereafter, we present the training exercises needed for smooth implementation of the initiative. Furthermore, a detailed explanation of how the team implemented the project, including the strategies adopted by the implementing partners and civil society, is described. The accomplishments of the Ghana initiative are delineated, accompanied by challenges encountered by partners, notably the lack of acceptance by certain service providers, particularly psychiatrists. Finally, we end with a discussion on how these challenges were overcome, the lessons learned and the way forward.

## Implementation partners

Eleven partners were involved in the implementation of the QualityRights initiative in Ghana. The Mental Health Authority, the Ministry of Health agency responsible for mental health services, served as the project lead. The WHO operates at all three levels of operations: Geneva Headquarters, the African Regional Office in Brazzaville and the Ghana Country Office in Accra. The Ghana Health Service, which is the largest agency of the Ministry of Health, has presence in all parts of Ghana. The Christian Health Association of Ghana, the second-largest agency of the Ministry of Health of Ghana, is made up of Christian churches and organisations with hospitals in underserved areas of Ghana.

Others are non-governmental organisations that work with persons with psychosocial disabilities, following a rights-based approach and the principles outlined in the CRPD. These are MindFreedom Ghana, Mental Health Society of Ghana, BasicNeeds Ghana, Ta-Excel Foundation, Inclusion Ghana, Special Olympics Ghana and Passion for Total Care.

## Method

### Inception, planning and coordination

In 2016, WHO Geneva Headquarters engaged with the MHA and several non-governmental organisations (MindFreedom Ghana and Mental Health Society of Ghana) to discuss an interest in implementing the QualityRights initiative on a nationwide scale. After consensus, the initial strategy identified partners who had worked with MHA and invited them to a 3-day sensitisation workshop. This workshop formed the project coordinating team in the MHA, with the Chief Executive Officer as the Chair and focal person. This team was tasked with overseeing the implementation of the initiative. A National Coordinator was appointed and a media consultant for traditional and social media campaigns was engaged. All three levels of the WHO were present at the meeting facilitated by WHO Headquarters in Geneva.

The team devoted the first 2 days to the development and adoption of strategies to ensure the successful implementation of the initiative. This was followed by a larger stakeholder meeting for the various strategies for discussion and agreement on the third day, involving 64 organisations. These included the Department for International Development (now the Foreign Commonwealth and Development Office), the Ministry of Health, the International Organization for Migration and STAR Ghana. Others included the psychiatric hospitals in Accra, Pantang and Ankaful.

The official launch of the QualityRights initiative in Ghana came off in February 2019 at the Accra International Conference Centre, attended by 800 people.

### Trainer of trainers workshop

After the launch, the implementing partners nominated people from their organisation to become QualityRights trainers. The team collaborated with foreign and local experts to train 108 participants (see Table 1 below) from implementing partners as trainers of trainers (ToTs). These ToTs trained others on the initiative by using a face-to-face approach across the country. This module of training was utilised because, among other benefits, it enhanced the capacity of trainers to deliver consistent, high-quality training to a larger audience within a relatively short time. This leads to more effective dissemination of knowledge, skills and best practices. A pool of facilitators was trained as ToTs to cascade the training downstream in their respective areas of operations. This was done for local buy-in to ensure efficiency and reduce cost. The ToT workshop started with the partners’ training, where three international consultants trained 26 people from the QualityRights implementing partners. For the ToT workshop, Ghana also collaborated with international partners. The University of Cagliari sent two observers to the ToT workshop. QualityRights Ghana partners trained other trainers in national and regional workshops.

**Table 1:** Distribution of trainer of trainers zonal and partners training

Training	Female	Male	Total
Partners	11	15	26
National	5	19	24
Northern Zone	8	19	27
Southern Zone	15	16	31
Total	39	69	108

Among the trainers were four people with lived experience and mental health patient support groups to share their experiences on human rights abuses.

### Nationwide training: two approaches

Nationwide QualityRights training efforts were conducted through the widescale rollout of e-training as well as in-person training workshops.

### WHO QualityRights e-training on mental health, recovery and community inclusion

The WHO QualityRights initiative provided a website specifically designed for Ghana, where learners could easily access and register for the WHO QualityRights e-training on mental health, recovery and community inclusion. The e-training consists of five core modules (understanding human rights; promoting human rights in mental health; realising recovery and the right to mental health and social services; protecting the right to legal capacity in mental health and social services; and creating mental health and related services free from coercion, violence and abuse) and two advanced case studies on legal capacity and ending coercive practices. On average, the training required no more than 18 h to complete. Participants could start and complete the programme at their own pace and place of convenience. A certificate was issued to a participant upon successful completion of all modules. Participants from the QualityRights Ghana partner organisations who completed the course shared their certificates with their organisations for the purpose of monitoring the achievements of the initiative's set targets.

In addition, internet-related challenges meant that some partners found it strategic to gather trainees to a central place where the internet reception was good, and offered free data for the training. This strategy increased the potential of the e-training to reach large numbers of interested individuals across the country.

The QualityRights e-training on mental health, recovery and community inclusion can be accessed for free by following this link: https://www.who.int/teams/mental-health-and-substance-use/policy-law-rights/qr-e-training.

From the period when the website went live to the time of writing, 40 443 people had registered for the training, 25 416 had started the training and 20 865 people had completed the training and obtained a certificate.

### In-person training

The 5-day in-person training for professionals and persons involved in the care of individuals with psychosocial, intellectual or cognitive disabilities consisted of the five core QualityRights training modules (human rights; mental health, disability and human rights; legal capacity and the right to decide; recovery and the right to health; freedom from coercion, violence and abuse).^13^ One full day of training was committed for each module. On average, trainees were kept to around 35 people within a training session to enhance interaction, and with the onset of COVID-19, all related protocols were implemented.

Regarding facilitation, usually four regional facilitators, including a national trainer, were available, plus the national coordinator, who was also a national trainer. The delivery approach was a structured Microsoft PowerPoint presentation with interactive sessions of group work, discussions and plenary sessions. The trainees sat in groups of six in a spacious room to allow for group work and social distancing. As of 31 December 2021, the team had facilitated 27 in-person trainings with 910 participants trained so far (see Table 2 below). It is worth noting that one of the partners (Mental Health Society of Ghana) used a patient organisation to conduct face-to-face training for selected patients across the regions of Ghana. The modules were adapted specifically for the patients and their caregivers to ensure it was practical and relevant to their situation. This resulted in a large turnout of patients for the in-person training.

**Table 2:** In-person QualityRights training

	Male	Female	Total
Patients	99	46	145
Commission on Human Rights and Administrative Justice	15	5	20
Social welfare	16	6	22
Health tutors	41	17	58
Health workers	283	173	456
Ghana education service tutors	5	3	8
Livelihood Empowerment against poverty	3	3	6
Police	12	5	17
Members of partner organisations	15	11	26
Traditional and faith-based healing centres	94	58	152
Total	583	327	910

### Implementation phase

There were three aspects of the e-training implementation phase:

- Initiatives to encourage interested persons who have been introduced to the programme to complete the e-training.
- e-training facilitated or sponsored by the partners: a partner would arrange a venue and provide data to motivate people to register, start, continue and finish the e-training. Individuals who successfully completed the e-training shared their certificates with the sponsoring partner so they could monitor the achievement of set targets for people completing the e-training.
- In-person training: before participation, learners were required to complete the e-training to build a solid foundation for the comprehensive face-to-face training and to enhance the achievement of the training objectives.

### Partner strategies

All of the implementing partners adopted various strategies to recruit trainees, motivating them to register and complete the e-training. Some partners published information on QualityRights in educational institutions with the help of tutors and student leaders by providing coaching for target segments to register and complete the QualityRights e-training. Another strategy took advantage of other public gatherings to introduce QualityRights e-training and encouraged the audience to register. They started with their own staff, followed by their operational communities, allies, and organisational partners and stakeholders.

### Partner and coordination meetings

Partners held regular fortnightly meetings to share strategies and evaluate their performance against agreed targets. The meeting encouraged partners who met their targets to keep working toward achieving their overall objectives. The meeting also supported those who had difficulty meeting targets, and encouraged them to revise and consider adopting strategies that had worked for other partners. These regular meetings helped to avoid duplication of efforts in accessing target groups and to ensure complementary efforts. As of 31 December 2021, there had been 37 such meetings, with an average attendance of 20 people.

### Resources for implementing partners

All partners who took part received support in resources. The team distributed 12 laptops, 17 desktop computers and 26 routers among the participating partners. This was to ensure support for logistical sufficiency in project implementation.

### Continuous professional development

As another strategy, the team got the professional regulatory bodies – namely the Medical and Dental Council, Nursing and Midwifery Council, Pharmacy Council and Allied Health Professional Regulatory Council – to accept the QualityRights training (both the e-training and the in-person training) as valid for the award of Continuous Professional Development credit points for renewing professional licences (see Table 3 below). This encouraged doctors, nurses, pharmacists and other professionals to complete the training. The project also encouraged teachers to use QualityRights modules for their training. At all events, meetings and other public functions, mental health professionals found space to bring attention to the QualityRights e-training and provided a website link to invite and recruit people to do the course.

**Table 3:** Professional accreditation of QualityRights training

Number	Professional institution	Continuous Professional Development points
1	Allied Health	e-training: 5 credits in-person 7 credits awarded
2	Psychology Council	3 credits awarded
3	Medical and Dental Council	3 credits awarded
4	Nursing and Midwifery Council	3 credits awarded
5	Pharmacy Council	3 credits awarded
6	Ghana College for Physicians and Surgeons	In process
7	Ghana College for Nurses and Midwives	Incorporated into their curriculum as a semester course

### Infusing QualityRights into school classroom lessons

The team made efforts to introduce the QualityRights initiative into the school curricula of secondary and tertiary institutions. An innovative way to encourage students to undertake the QualityRights e-training exercise, particularly at tertiary institutions, involved a model where teachers introduced the e-training to the class and encouraged students to sign in and complete the training. For instance, an IT teacher found a way of introducing the e-training into the curriculum by introducing his class with a free lesson in IT and asking students to complete the e-training for their computer lessons.

### Promotion of QualityRights on social media

To take full advantage of the benefits that social media offers and the potential to reach more people with QualityRights e-training, we formed a QualityRights media team to ensure effective publicity of the initiative in Ghana. The team developed a QualityRights website, which featured endorsement videos of selected QualityRights champions speaking agreed messages, including testimonials of beneficiaries of the e-training. One could sign up to receive routine updates, upcoming events and success stories of the impact of the initiative. The site used a media blog, which contained news, event updates, galleries and videos. The team linked the various MHA social media handles (Twitter, Instagram, Facebook and YouTube) to widen their reach. These handles received appreciable followers and impressions.

### Google ads campaign

To encourage more people to register and engage with the QualityRights website, the team created two Google Ads campaigns leading to the website or the e-training landing page. This has been running since May 2019. The Google ads targeting the e-training website recorded 2380 clicks and 330 000 impressions; those targeting the QualityRights website recorded 1640 clicks and 171 000 impressions as of the first year of implementation. The team created and sent 2000 e-newsletters to verified email addresses containing a brief about the QualityRights initiative in Ghana, opportunities for volunteers and an e-training link to the website.

### Targeted text messaging

The team generated 5700 text messages containing promotional messages for the QualityRights initiative in Ghana. The team sent these to professionals in the major cities of Ghana, encouraging them to register.

### ‘Digital Angels’

Finally, the team came up with a concept known as ‘Digital Angels’. This comprised volunteers who signed up through newsletters to champion QualityRights -e training in Ghana. This concept attracted 120 volunteers recruited through other social media platforms and taken through e-training. Some of the ‘Digital Angels’ are social media influencers and bloggers who featured promotional stories on their blogs and tweeted promotional contents.

### QualityRights champions

Another strategy used was the development and production of one-minute video messages that would resonate with the targeted audience. These videos featured 25 prominent Ghanaians and other personalities identified as QualityRights champions. The team considered these personalities as influencers in the Ghanaian society and appropriate to associate with the project, thus giving greater identity to the initiative. These included a former Deputy Director-General of the WHO, the WHO Country Representative the Deputy Minister for Health, the Chief Executive of the MHA and a person with lived experience of psychosocial, intellectual or cognitive disability. The team aimed these messages at protecting, promoting and fulfilling the rights of persons with psychosocial, intellectual or cognitive disabilities, to encourage the elimination of all kinds of abuses against vulnerable individuals. These video messages featured prominently on the dedicated website and other social media platforms, such as Facebook and Instagram.

### Financing the QualityRights initiative

The WHO funded the initiative through funds from the Fondation d'Harcourt of Switzerland, the Foreign and Commonwealth Development Office (FCDO) of the German Government, and the European Union. The European Commission contributed funding through a grant within the ‘European Instrument for Democracy and Human Rights (EIDHR): 2017 Global Call for Proposals’ and the project ‘Empowering Persons with Psychosocial Disabilities to Fight for Their Rights: An Implementation of the CRPD and QR Principles in Ghana, Lebanon, and Armenia’ led by the University of Cagliari, Italy.

## Results and discussion

### Acceptance and achievements

On 9 November 2020, the Director-General of the WHO acknowledged the performance of Ghana's QualityRights Initiative at the World Health Assembly. At the time of writing this paper, 40 443 people had registered for the e-training, 25 416 had started the e-training and 20 865 people had completed the training and been awarded a certificate. Furthermore, the team had facilitated 27 in-person training courses, and 910 participants had trained and received their certificates**.** Reports received from some service providers during the mental health annual performance review indicate that the quality of service they now provide has improved because of the training. In the same way, patients who have undergone QualityRights training report feeling empowered to contribute to their recovery in patient satisfaction surveys conducted by psychiatric hospitals. For instance, a hospital director at the national mental health performance review for 2020 pointed out that most staff had undergone QualityRights e-training and added that seclusion had gone down markedly in 2019, the year under review.

### What worked: supportive factors

#### Quality of partners

There were some supportive factors that contributed to the success of the programme, mainly through feedback from participants who provided pre- and post-test questionnaires and testimonials. Key among them was the quality of the partners that were selected to be part of this project. These were partners who worked very hard and were highly committed to the cause of improving the quality of care and implementing a rights-based approach to mental health in Ghana.

### Strong leadership team

The MHA, as the lead agency in the QualityRights implementation, provided strong leadership to ensure cohesion and unity of purpose. The National Coordinator, who was also a staff member of the MHA, was active in organising and coordinating meetings, effective in monitoring progress and available to provide support to partners.

### Effective partnership and collaboration

There was commitment from all of the partners and collaborators. The team held a series of virtual meetings between the three levels of the WHO and the partners in Ghana, with the WHO providing support, guidance and funding. Ghana also collaborated with the Ministry of Public Health, Lebanon; the Armenian Psychiatric Association; the Ministry of Health, Kenya; Cagliari University, Italy; the Special Olympics Africa and others to implement the project internationally.

### Motivation to partners

The team supported partners with equipment, including laptops, mobile data for e-training and funds for paperwork and transportation cost reimbursement. This support and motivating incentives contributed to the commitment of partners.

### What did not work

The national/master trainers’ team had only one psychiatrist, who is also the lead author. At a face-to-face training for psychiatrists, the trained psychiatrist was not available. This presented a major challenge, as the psychiatrists felt the facilitators did not address their concerns. It also emerged at a consultative workshop that the initiative has not been adapted to local circumstances, as the examples and scenarios provided were foreign. For instance, the law in Ghana, like other jurisdictions, allows for the involuntary admission of a person in circumstances of severe mental illness where the person is a danger to themselves, others or property; however, the initiative did not recognise this provision.

### Non-acceptance and other challenges

Although stakeholders had accepted the initiative, there have been a few challenges. First, although the QualityRights initiative presents an opportunity to transform human rights conditions in mental healthcare, the three major psychiatric hospitals are under-resourced financially and logistically, a situation that blocks quality care in line with human rights standards suggested by the initiative. Second, the cost of the data needed, coupled with poor internet connectivity in some areas, was reportedly frustrating and caused some people to drop out. Third, the COVID-19 pandemic was a major challenge to the rate of implementation of the project. The pandemic, with its lockdown and movement restrictions, forestalled physical coordination meetings for planning and logistics. It also led to the suspension of the in-person trainings and slowed the implementation of the project. Finally, there was an initial non-acceptance by some psychiatrists and clinical psychologists. This non-acceptance is because the Mental Health Law, Act 846 (2012), makes provision for involuntary admission in circumstances where a person is believed to be suffering from severe mental illness. However, it seems the initiative made no such provisions. Furthermore, concerns about the marginalisation of psychiatry – similar to the antipsychiatry arguments by Hoare and Duffy^14^ – came up during sessions. This required a special session to clear up the misconceptions, using the arguments by Moro et al^15^ as the basis. They have argued that the criticisms notwithstanding, psychiatrists have an essential role to play in promoting human rights in mental healthcare, and the WHO QualityRights initiative is a perfect tool to achieve this objective. In addition, there was constant engagement with relevant stakeholders to explore alternatives to involuntary treatment as much as possible, using approaches such as advance directives. Most importantly, it was recommended that this be considered in adapting and contextualising the initiative in Ghana for future training.

### The way forward

With reference to the four areas stated by Funk and Drew,^1^ it can be concluded that the initiative in Ghana has been successful. Furthermore, several other factors contributed to the successful implementation of the initiative. These include teamwork, partnership among participating stakeholders, motivation, strong leadership, the acceptance of the initiative and the outcome**.** The collaboration with the WHO and other partners contributing to implementing the QualityRights initiative has witnessed sustainable positive changes in the attitudes of stakeholders. Although some participating partners had some challenges during implementation, the team considered new strategies to overcome the challenges and ensure sustainability beyond the project life cycle. These new strategies include arranging for more credit points in Continuous Professional Development for QualityRights training. The team encouraged heads of institutions to support students with internet data so they could complete the e-training. Some partners have considered funding through grant proposals to continue the project beyond its current lifespan. Finally, some partners intend to encourage school authorities to allow the training to take place during their normal school hours at no extra cost to the facilitating organisations.

Ghanaians, having gained expertise in QualityRights, are now training people outside of Ghana (i.e. Kenya, Zimbabwe and Italy, a high-income country), sharing the lessons learned and supporting mental health reform abroad.

## Data Availability

The data that support the findings of this study are available from the corresponding author, A.O.O., upon reasonable request.
